# The Utility of Immature Granulocyte Count and Percentage on the Prediction of Acute Appendicitis in the Suspected Acute Appendicitis According to the Alvarado Scoring System: A Retrospective Cohort Study

**DOI:** 10.5152/tjg.2022.21865

**Published:** 2022-10-01

**Authors:** Özlem Güler, Mehmet Buğra Bozan, Filiz Alkan Baylan, Serdar Öter

**Affiliations:** 1 Department of Emergency Medicine, Kahramanmaraş Sütçü İmam University Faculty of Medicine, Kahramanmaraş, Turkey; 2 Department of General Surgery, Kahramanmaraş Sütçü İmam University Faculty of Medicine, Kahramanmaraş, Turkey; 3 Department of Biochemistry, Kahramanmaraş Sütçü İmam University Faculty of Medicine, Kahramanmaraş, Turkey; 4 Department of Gastroenterologic Surgery, Manisa State Hospital, Turkey

**Keywords:** Acute appendicitis, computerized tomography, immature granulocyte count, immature granulocyte percentage, Alvarado score

## Abstract

**Background:**

This study aimed to investigate the utility of immature granulocyte count and percentage on the prediction of suspected acute appendicitis according to the Alvarado scoring system and its effect on the need for computed tomography scanning.

**Methods:**

Adult patients who had an Alvarado scoring system between 4 and 7 with the first imaging technique computed tomography were included and retrospectively analyzed. The immature granulocyte count and granulocyte percentage were obtained from the blood samples taken at the time of the patient’s first admission to the hospital.

**Results:**

A total of 652 patients were evaluated and 186 patients were included in the study. Acute appendicitis was not detected in computed tomography imaging of 121 (65%) patients (group N) and detected in 65 (35%) patients (group P). The mean immature granulocyte percentage in group N and group P were 0.314 ± 0.188 (0.00-1.40) and 0.364 ± 0.205 (0.05-1.00), respectively. The mean immature granulocyte percentage was similar between groups (*P* = .095). The mean immature granulocyte count was 33 ± 46/µL (0-50) in group N and 60 ± 85/µL (10-690) in group P. Immature granulocyte count was significantly higher in group P (*P* = .005). Univariate analysis results revealed that age and immature granulocyte percentage were not predictive factors for the presence of acute appendicitis in suspected cases (*P *> .05). On the other hand white blood cell, neutrophil–lymphocyte ratio, C-reactive protein, and immature granulocyte count were determined as predictive factors in univariate analysis and multivariate analysis. Receiver operating characteristic curve analysis of preoperative immature granulocyte percentage and immature granulocyte count values in the diagnosis of acute appendicitis: the cut-off value of immature granulocyte percentage was ≥0.35 and its sensitivity, specificity, positive predictive value, and negative predictive value were 44.1%, 72.1%, 71.1%, and 41.5%, respectively (area under the curve: 0.588; CI: 0.484-0.682). The cut-off value of immature granulocyte count was ≥35/µL and its sensitivity, specificity, positive predictive value, and negative predictive value were 66.1%, 73.6%, 71.9%, and 67.7%, respectively (area under the curve: 0.743; CI: 0.659-0.827)

**Conclusion:**

Immature granulocyte count is a predictive factor for acute appendicitis in patients with the middle-risk group according to the Alvarado score and may be useful for the selective use of tomography.

## Main Points

- There are too many clinical and imaging modalities used for the identification of acute appendicitis (AA); however, all the procedures have missed areas like the Alvarado scoring systems suspended part.
- The immature granulocyte count in automated complete blood cell calculation added to the Alvarado scoring system can diminish the complicated AA case rates due to delayed diagnosis and increase the correct diagnosis rates.
- In rural areas, immature granulocyte count in routine hematologic tests can be a helpful diagnostic test for the suspected AA cases according to the Alvarado scoring system (scores 4-7).
- In the suspected abdominal pain cases according to the Alvarado scoring system (scores 4-7) adds additional test costs (e.g., computed tomography) and the Immature granulocyte count automatically calculated in routine hematologic tests can diminish the need for diagnostic additional tests.

## Introduction

Acute appendicitis (AA) is the most common cause of abdominal pain requiring surgery in the emergency department.^1^ The highest incidence of AA is in the second and third decades of life, but it can be seen at any age. There is a 7% chance that a person will have appendicitis during life. Only half of the patients with AA are presented with typical periumbilical pain followed by nausea, vomiting, and the migration of pain to the right lower quadrant.^2,3^ The diagnosis of AA is based on the patient’s medical history, physical examination, and laboratory findings. Various clinical prediction rules have been developed to increase diagnostic accuracy. The most accepted among these is the Alvarado scoring system (ASS).^4^ Alvarado scoring system classifies patients as low, moderate, and high-risk groups for AA (Table 1). Accordingly, it recommends discharge, observation, and surgical intervention to patients. However, such scoring systems should not be used as the only method in diagnosis.^5^

Increased imaging use in patients with suspected AA improved the rate of correct diagnosis. American College of Radiology Appropriateness Criteria (ACR) recommends computed tomography (CT) as the primary imaging method to confirm the diagnosis of AA in adults.^6,7^ However, CT imaging has some disadvantages, such as radiation exposure, undesirable effects associated with the use of contrast agents, additional cost, and increased workload in the emergency room.

Numerous inflammatory parameters adapted from complete blood cell count (CBC) can be used in infectious inflammatory processes such as AA, pyelonephritis, and non-infectious conditions such as differentiation of tumoral masses from benign lesions and determining survival in acute myocardial infarction.^8-11^ Among these parameters, neutrophil–lymphocyte ratio (NLR), platelet–lymphocyte ratio, and lymphocyte–monocyte ratio were used in previous studies. Similarly, the count of immature granulocytes (IGC), which is an indicator of increased activation of the bone marrow, and the percentage of IG (IGP), which is the ratio of IGs to the total white blood cell count (WBC), are also has been used in acute necrotizing pancreatitis, differentiation of complicated AA from uncomplicated AA, pyelonephritis, sepsis, thyroid gland malignancies, and renal cell carcinomas.^9,12-14^ Previously, the determination of the number of IGs could be possible based on counting granulocyte precursor cells during the direct microscopic examination.^10,11^ Nowadays, automatic blood analyzers can easily measure the amount and percentage of IGs simultaneously in a complete blood count test with advances in technology.^15^ The IGs in peripheral blood are an indicator of increased bone marrow ­activity.^12^ It has been reported in previous studies that IGC and IGP increase during infection and sepsis and are more reliable markers in the diagnosis of AA than other hematological parameters.^9,13^

This study was aimed to investigate the utility of IGC and IGP on the prediction of suspected AA according to the ASS and its effect on the need for CT scanning.

## Materials and Methods

### Study Design and Setting

This study is a retrospective cohort analysis involving adult patients who were admitted to the emergency department of Kahramanmaraş Sütçü İmam University Hospital, between January 2019 and July 2019 due to abdominal pain and suspected AA according to ASS. After approval of the Clinical Trials Ethical Committee of the Medical Faculty of Kahramanmaraş Sütçü İmam University (Date: 19.06.2019; Session Number: 2019/11; Decision No: 06) all the data of included cases were retrospectively evaluated. Because of the retrospective character of the study design, the signed informed consent of patients is not required. All procedures performed in studies involving human participants were under the ethical standards of the institutional and/or national research committee and with the 1964 Declaration of Helsinki and its later amendments or comparable ethical standards.

### Patient Selection and Data Collection

Adult patients who applied to the emergency department of Kahramanmaraş Sütçü İmam University Hospital due to abdominal pain and had an ASS between 4 and 7 and whose initial imaging technique was CT were included in the study. Alvaradoscores of the patients were noted from the patients’ medical records. Patients were used other imaging methods, underwent surgery without imaging, followed by medical treatment without surgery, pregnant, under the age of 18, with incomplete medical records, and with additional diseases (such as underlying hematologic or rheumatologic disease, other concurrent infectious diseases) and treated with granulocyte colony-stimulating factors, glucocorticoids, or other immunosuppressants that may affect inflammation markers were excluded from the study.^13^

Electronic files of the patients recorded in the hospital database were reviewed by the authors. Demographic data (age and gender), laboratory values, tomography reports, ASS scores, and pathological diagnoses were recorded. The diagnosis was also confirmed histopathologically in all patients diagnosed with AA according to the result of tomography.

White blood cell, neutrophil count, lymphocyte count, IGC, and IGP were measured using an automated hematological analyzer (XN 3000; Sysmex Corp., Kobe, Japan). The neutrophil–lymphocyte ratio was calculated manually. The patients were divided into 2 groups as those with AA (appendicitis positive group, group P) and non-appendicitis (appendicitis negative group, group N) according to the result of tomography.

### Statistical Analysis

The data were analyzed with the IBM Statistical Package for the Social Sciences (SPSS version 20) program (IBM Corp.; Armonk, NY, USA). The compliance of continuous variables to normal distribution was evaluated with the Shapiro–Wilk test. An independent sample *t*-test was used to compare the data that complied with the normal distribution and the Mann–Whitney *U* test was used for those that did not comply with the normal distribution. Pearson test was used for the correlation analysis of normally distributed parameters, and the Spearman test was used for correlation analysis of non-normally distributed parameters. Multivariate analysis was performed with parameters found to be significant in the evaluation of univariate analysis and predictive values of preoperative blood parameters were calculated. The chi-square test was used to analyze categorical variables. *P *< .05 value was considered significant. The receiver operating characteristic (ROC) curve analysis was used to evaluate the success of laboratory parameters in predicting AA diagnosis. The area under the curve (AUC), sensitivity, specificity, positive predictive value (PPV), negative predictive value (NPV), and accuracy rate were calculated for parameters with a statistically significant difference between groups.

## Results

**Figure 1.**
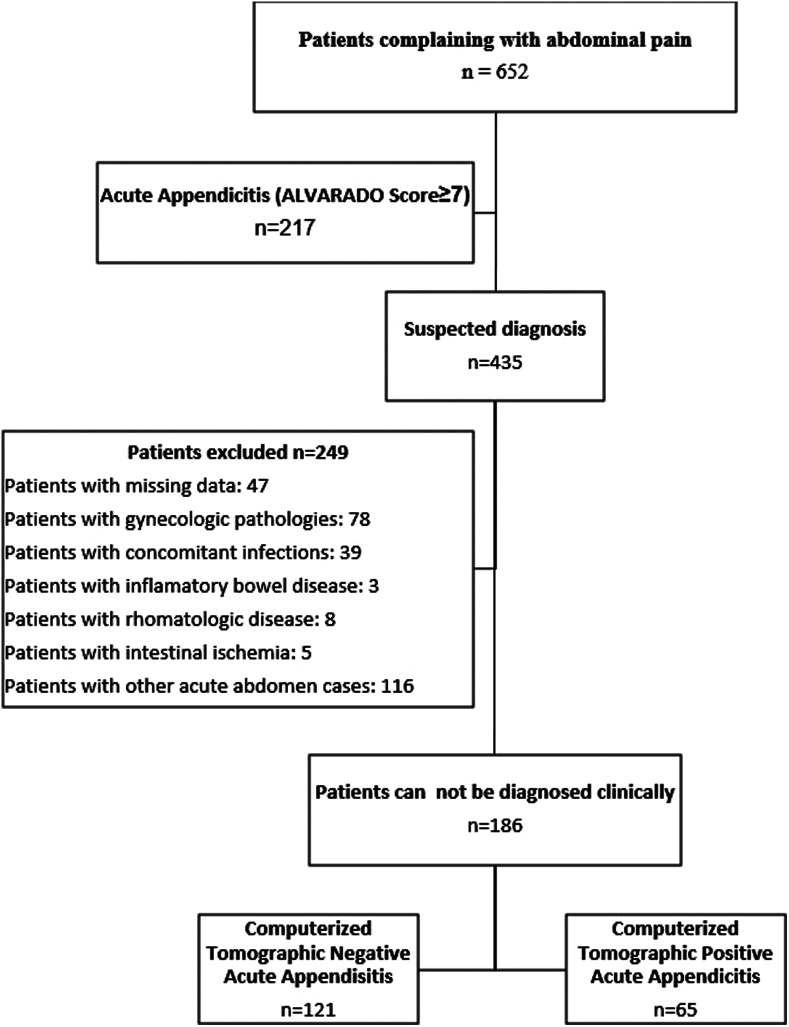
Flowchart of the study design.

A total of 652 patients complaining of abdominal pain were evaluated retrospectively and 186 patients who met the inclusion criteria were included in the study (Figure 1). Acute appendicitis was not detected in CT imaging of 121 (65%) patients and 76 (63%) of these patients were women and 45 (37%) were men. Sixty-five (35%) patients diagnosed with AA on CT imaging; 27 (42%) were female and 38 (58%) were male. The mean age in group N was 39.80 ± 16.53 (19-84), and the mean age in group P was 37.38 ± 15.24 (18-88) (*P *= .330). The hematological and biochemical parameters of the groups and their comparison results are presented in Table 2.

The mean IGP in group N and group P were 0.314 ± 0.188% (0.00%-1.40%) and 0.364 ± 0.205% (0.05%-1.00%), respectively (*P *= .095). The mean of IGC in group N was 33 ± 46/µL (0-50) and 60 ± 85 /µL (10-690) in group P (*P *= .005).

**Figure 2.**
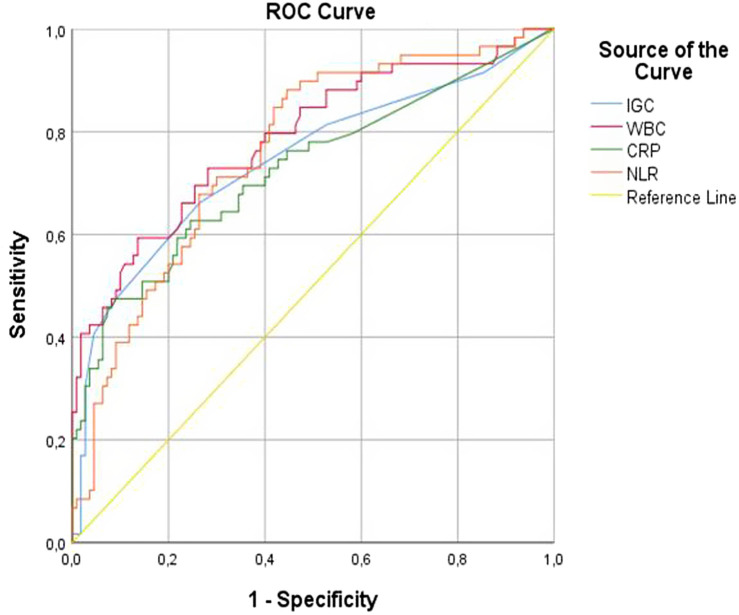
Receiver operating characteristic (ROC) curve analyses of IGC, WBC, CRP, NLR, IGC, and IGP to predict acute appendicitis on tomography. (IGC, immature granulocyte count; WBC, white blood cell; CRP, C-reactive protein; NLR, neutrophil to lymphocyte ratio)

Univariate analysis results revealed that age (*P *= .330; F =0.952; odds ratio [OR] = 0.005) and IGP (*P *= .095; F = 2.817; OR = 0.015) were not seen as predictive factor for the presence of AA in suspected cases according to ASS. On the other hand WBC, NLR, CRP, and IGC were determined as predictive factors in univariate analysis. In multivariate analysis, WBC, NLR, CRP, and IGC parameters were found to be predictive factors of AA in clinically suspected cases according to ASS in the preoperative period (Table 3). The ROC curve analysis of preoperative IGP and IGC values in the diagnosis of AA in group P was given as follows: the cut-off value of IGP was ≥0.35% and its sensitivity, specificity, PPV, and NPV were 44.1%, 72.1%, 71.1%, and 41.5%, respectively (AUC: 0.588; CI: 0.484-0.682); the cut-off value of IGC was ≥35/µL and its sensitivity, specificity, PPV and NPV were 66.1%, 73.6%, 71.9%, and 67.7%, respectively (AUC: 0.743; CI: 0.659-0.827) (Figure 2 and Table 4).

## Discussion

Acute appendicitis is the most common cause of abdominal pain requiring urgent surgery in the emergency department.^16^ Appendicitis is an inflammatory process that can result in perforation, abscess formation, general peritonitis, bowel obstruction, and rarely, 0.08% death. In the case of perforation, mortality risk rises to 0.5%.^17^ Therefore, it is of utmost importance to correctly diagnose AA.^18^ Although the use of CT is widespread today, the diagnosis of AA is still based on symptoms, findings, and laboratory results, especially in rural areas where additional imaging methods are insufficient.^18^

Acute appendicitis can be easily diagnosed in its classic form, but classical presentation occurs in 50%-60% of patients. Atypical presentations are most common in conditions such as anatomical location variation of the appendix, extreme ages, and pregnancy.^19^ Symptoms may vary from person to person or ambiguous abdominal discomfort is the only symptom in the early stages of inflammation. Many patients with atypical clinical manifestations are diagnosed with complicated appendicitis.^20^ Therefore, timely and accurate diagnosis in the emergency department remains a clinical challenge in the early stages or atypical cases. Despite all the improvements in diagnosis and treatment, high-negative appendectomy and perforation rates are still reported (13%-36% and 12%-21%, respectively).^9^

Alvarado scoring system is the most widely used scoring system for the diagnosis of AA. For patients ASS with ≤4 discharge, 24-hour observation for patients with ASS 5-7, and surgery for patients with ASS 8-10 were recommended in many studies.^4^ The diagnostic suitability of the ASS was determined as 90.9% for 7-10 points and 100% for 0-4 points.^17^ One hundred eighty-six patients, including 103 women and 83 men with ASS 4-7 who underwent CT imaging were included in our study. Acute appendicitis was diagnosed in 34.9% of the patients and the ratio of males to females was 1.4:1. The lifetime incidence of AA was 8.6% in males, 6.7% in females, and the ratio of males to females was 1.4:1, 1.5:1, 2:1.4 in different studies.^21^ There are many gynecological diseases (such as ovarian cyst rupture and ovarian torsion) especially in young adult female patients that may be confused with AA.^22^ Therefore, conditions with atypical presentations are more common in women.

Many studies have been conducted in the literature about the usefulness of laboratory tests to support the diagnosis of AA. The most frequently studied parameters are WBC, CRP, neutrophil rate, procalcitonin, erythrocyte sedimentation rate, NLR, and bilirubin.^9,20^ Among them, serum CRP and WBC are widely used in the diagnosis process of patients with suspected appendicitis in the emergency department.^23^ Predictive values of the CRP and the count of WBC were investigated in patients with AA in the study of Beltran et al.^24^ The sensitivity of the WBC count at the 12th hour after symptoms started was 90% according to their study. They found that CRP values remained high at the 12th, 24th, and 48th hours after symptoms started.^24^

Kharbanda et al.^25^ showed that CRP is more beneficial in children with pain from 24 to 48 hours to predict appendicitis and WBC in those with less than 24 hours of pain. The WBC count is an early marker of appendix inflammation, but it cannot reliably distinguish acute and perforated appendicitis. C-reactive protein has been shown to increase significantly after appendix perforation or abscess development.^26^ White blood cell and CRP levels were not detected as a predictive factor for perforation in AA in another study conducted by Güler et al.^27^ In our study, we determined the cut-off value of CRP to be 7.30 mg/L to predict the diagnosis of AA in moderate-risk patients according to ASS, and appendicitis was shown in CT imaging.

Shin et al.^13^ found that NLR ≥ 5.7 had 57.2% sensitivity and 80% specificity as a positive predictor for an appendectomy. Increased NLR levels were shown to be an effective predictive factor in diagnosing AA and detecting complicated AA.^28^ In a study of 112 patients conducted by Akgül et al.^29^ the NLR value was determined as a predictive factor for the diagnosis of AA. For the diagnosis of AA, we detected sensitivity, specificity, PPV, and NPV at 3.77 cut-off value as 70.8%, 69.4%, 70.41%, and 70.2%, respectively.

There are discussions about the usefulness of the IGP in predicting AA. A study conducted by Shin et al.^13^ showed that the sensitivity for the 0.2 cut-off value for IGP was reported as 59.8% and specificity 77.1%. With these values, it was reported that IGP may be useful in predicting the diagnosis of AA.^13^ However, Park et al.^20^ reported that IGP did not provide an additional contribution for 0.3 cut-off value in the exclusion of AA (sensitivity: 54% and specificity: 56%). In our study, AUC, sensitivity, specificity, and *P* values were found to be 0.575, 42.5%, 71.1%, and .093, respectively for a 0.35 cut-off IGP value. Our findings show that IGP will not be useful in supporting the diagnosis of AA in clinically suspected patients according to ASS.

In a study conducted by Ünal et al.^9^ for the cut-off value 60.5/µL of IGC, AUC, sensitivity, specificity in predicting AA was reported as 0.795, 55.9%, and 96.1%, respectively. In the same study, it was reported that IGC is superior to WBC, NLR, and IGP to distinguish acute simple appendicitis and acute complicated appendicitis.^9^ In our study, we determined the sensitivity, specificity, PPV, and NPV of IGC with a 35/µL cut-off value as 66.1%, 73.6%, 71.9%, and 67.7%, respectively.

The diagnostic accuracy for AA has increased with the use of CT. Cost analysis studies conducted after the ACR recommended tomography as the most appropriate test for working in the right lower quadrant pain showed that routine use of tomography in the evaluation of suspected appendicitis reduces healthcare costs. Also, routine tomography use has been found to significantly reduce negative appendectomy rates.^19^ However, there are some disadvantages associated with CT imaging, including complications related to radiation exposure and contrast agent use. Additionally, in rural areas, imaging modalities cannot be reached easily. For this reason, predicting AA with clinical and laboratory findings becomes more useful in rural areas.

The ASS is a well-established and widely used clinical decision tool. It can help in reducing the need for CT scanning to diagnose appendicitis. Tan et al.^30^ reported that ASS had a general sensitivity of 94.2% to exclude AA in patients with ASS 3 and below. Patients with ASS 7-10 for men and ASS 9-10 for women were recommended to undergo surgery without imaging. This application has been reported to reduce CT scan usage by 70%. However, when our patients were analyzed according to the recommendations of this study, tomography was negative in terms of AA in 65.1% of our 186 patients with ASS 4-7. As can be seen, negative tomography ratios remain high if only ASS is used as a guide when making CT imaging decisions. Our results show that the use of elevated IGC with ASS can be an easily applicable and predictive factor for AA in clinically suspected AA cases and can reduce the need for CT scan.

Our study has some limitations due to its retrospective design. The study was conducted in the emergency department of a single university hospital, and the cohort was relatively small. Laboratory parameters of the patients were obtained from the blood samples taken at the first admission to the hospital. Information about the onset of symptoms and the time of hospital admission was not available for all patients included in the study. Similarly, data of duration between diagnosis to operation time were not available. However, the strongest side of our study is being the first study evaluating the utility of IGC combined with ASS for the detection of AA in suspected cases and detection of CT scanning needs for these patients.

## Conclusions

Due to the uncertainty of its symptoms and the lack of diagnostic biomarkers, early and accurate diagnosis of AA is still a challenge. Early recognition and treatment of AA are critical to prevent complications such as abscess development and perforation. Immature granulocyte count is a predictive factor for AA in patients with the moderate-risk group according to the ASS score, which can be easily measured with routine CBC examination without additional cost and may be useful for the selective use of tomography.

## Figures and Tables

**Table 1.** Alvarado Scoring System for Acute Appendicitis

**Parameters**	**Score**
**Symptoms** Migratory right iliac fossa pain Anorexia Nausea/vomiting	1 1 1
**Signs** Right lower quadrant tenderness Right iliac fossa rebound Elevation of temperature	2 1 1
**Laboratory** Leukocytosis Left shift (neutrophils)	2 1
**Total score**	10

**Low risk: **≤ 4; consider alternative diagnosis, these patients were considered to probably not have acute appendicitis.

**Moderate risk: **5-6; follow-up, choose proper treatment options after additional diagnostic approaches

**High Risk: **≥7; highly probably appendicitis, choose proper treatment options for acute appendicitis (medical treatment, radiologic interventions, and appendectomy)

**Table 2.** The Hematological and Biochemical Parameters of the Groups and Their Comparison Results

**Parameter**	**Group-N** ** (n = 121)**	**Group-P** **(n = 65)**	*P*
**Age (years)**	39.80 ± 16.53 (19-84)	37.38 ± 15.24 (18-88)	.330
WBC (×10**3 /µL)**	9.25 ± 2.40 (3.61-15.70)	12.95 ± 3.83 (5.57-21.27)	<.001*
Neutrophils (×10**3 /µL)**	6.18 ± 2.29 (2.08-12.10)	10.10 ± 3.77 (3.23-18.65)	<.001*
Lymphocytes (×10**3 /µL)**	2.27 ± 0.99 (0.25-5.70)	1.91 ± 0.99 (0.49-5.26)	.019*
**IGC(/µL)**	33 ± 46 (0-50)	60 ± 85(1-69)	.005*
**IGP (%)**	0.314 ± 0.188 (0.00-1.40)	0.364 ± 0.205 (0.05-1.00)	.095
**NLR**	3.67 ± 3.17 (0.70-15.87)	7.34 ± 3.17 (1.17-32.62)	.001*
**CRP (mg/L)**	9.37 ± 11.96 (3.02-65.50)	39.98 ± 53.01 (3.02-280)	<.001*
**Glucose(g/dL)**	111.17 ± 26.74 (75-223)	115.18 ± 22.78 (79-209)	.307
**Urea (mg/dL)**	11.89 ± 3.76 (5-27)	11.64 ± 3.71 (3-24)	.677
**Creatinine (mg/dL)**	0.72 ± 0.17 (0.4-1.24)	0.93-1.41 (0.3-12)	.116
**AST (U/L)**	22.81 ± 11.32 (9-90)	19.56 ± 6.84 (4-45)	.036*
**ALT (U/L)**	21.51 ± 17.21 (7-169)	18.30 ± 7.93 (5-50)	.155
**Sodium (meq/L)**	139.20 ± 3.23 (117-145)	138.87 ± 2.18 (133-143)	.460
**Potassium (meq/L)**	4.27 ± 0.37 (3.18-5.30)	4.30 ± 0.39 (3.10-5.20)	.588
**Calcium (mg/dL)**	9.16 ± 0.46 (7.70-10.50)	9.17 ± 0.54 (7.16-10.70)	.892

*P < .05 is statiscally significant.

**WBC, **white blood cell; **IGC, **immature granulocyte count; **IGP, **immature granulocyte percentage; **NLR, **neutrophil to lymphocyte ratio; **CRP, **C-reactive protein; **AST,** aspartate aminotransferase; **ALT, **alanine aminotransferase.

**Table 3.** Multivariate Analyses Results of İnflammatory Markers for Prediction of Acute Appendicitis for Clinically Suspected Cases According to ASS

	**Odds Ratio**	**B**	*P*	**%95 CI**	
**Lower Bound**	**Upper Bound**
**WBC**	0.864	12.992	<.001*	12.205	13.778
**NLR**	0.508	7.052	<.001*	5.992	8.112
**CRP**	0.345	39.981	<.001*	31.563	48.398
**IGC**	0.241	0.062	<.001*	0.045	0.079

*P < .05 is statiscally significant.

**WBC, **white blood cell count; **NLR, **neutrophil to lymphocyte ratio; **IGC, **immature granulocyte count; **CRP, **C-reactive protein; **ASS, **Alvarado Scoring System.

**Table 4.** ROC Analysis of WBC, NLR, IGP, IGC, and CRP for Group P

**Parameters**	**ARUC**	**Asymptotic 95% CI**		*P*	**Sensitivity (%)**	**Specificity (%)**	**Cut-Off Value**	**PPV** **(%)**	**NPV** **(%)**
**Lower Bound**	**Upper Bound**
**WBC (/mm 3 )**	0.787	0.711	0.862	<.001*	72.9	71.2	≥10660	71.1	73.8
**NLR**	0.761	0.686	0.836	<.001*	71.2	70.0	≥3.77	68.6	70.8
**IGP (%)**	0.588	0.494	0.682	.059	44.1	72.7	≥0.35	71.1	41.5
**IGC (/mm 3 )**	0.743	0.659	0.827	<.001*	66.1	73.6	≥35	71.9	67.7
**CRP (mg/L)**	0.726	0.641	0.842	<.001*	69.5	64.5	≥7.29	64.5	69.5

*P < .05

**WBC, **white blood cell count;** NLR, **neutrophil to lymphocyte ratio; **IGC, **immature granulocyte count; **CRP, **C-reactive protein; **IGP, **immature granulocyte percentage; **AUC, **area under curve; **PPV, **positive predictive value; **NPV, **negative predictive value; ROC, receiver operating characteristic.
